# Associations between Parents’ Health Literacy and Sleeping Hours in Children: A Cross-Sectional Study

**DOI:** 10.3390/healthcare6020032

**Published:** 2018-04-02

**Authors:** Hiroto Ogi, Daisuke Nakamura, Masato Ogawa, Teruhiko Nakamura, Kazuhiro P. Izawa

**Affiliations:** 1 Department of Physical Therapy, Faculty of Health Sciences, Kobe University School of Medicine, 7-10-2 Tomogaoka, Suma-ku, Kobe 654-0142, Japan; ogihiroto6062@gmail.com; 2 Cardiovascular stroke Renal Project (CRP), 7-10-2 Tomogaoka, Suma-ku, Kobe 654-0142, Japan; nakadai525@gmail.com (D.N.); mogawa@med.kobe-u.ac.jp (M.O.); 3 Department of International Health, Graduate School of Health Sciences, Kobe University, 7-10-2 Tomogaoka, Suma-ku, Kobe 654-0142, Japan; 4 Division of Rehabilitation Medicine, Kobe University Hospital, 7-5-2 Kusunoki-cho, Chuo-ku, Kobe 650-0017, Japan; 5 Educational Corporation Tsukushi Gakuen, 2-3-11 Takadai, Chitose 066-0035, Japan; nakamura_00_00@yahoo.co.jp

**Keywords:** parents’ health literacy, sleeping hours, children

## Abstract

Background: Sleep in preschool children is an important factor for their health and active lives. The lack of adequate sleep in preschool children is a serious public problem in Japan. The relationship between health literacy (HL) and health status is well recognized. The purpose of this study was to investigate the association between the sleep duration of preschool children and the HL of their parents. Methods: In the present study, participants were preschool children (3–6 years) and their parents. We assessed the HL of the parents with the 14-item Health Literacy Scale (HLS-14) questionnaire. Sleep duration of the children was reported by their parents. We divided parents into two groups according to HLS-14 score and analyzed children’s sleeping time separately. Results: Data from 279 parents and their children were ultimately analyzed. The high HL group comprised 210 families (75.3%) and the low HL group comprised 69 families (24.7%). Average children’s sleep duration was significantly longer in the high HL group (9.5 ± 0.9 h) than in the low HL group (9.1 ± 1.1 h) (*p* = 0.013). A positive correlation was found in the low HL group between parents’ HL and their children’s sleeping times (*p* < 0.01, r = 0.32) but the difference was not significant in the high HL group (*p* = 0.98, r = −0.0009). Conclusion: The HL of parents appears to affect their children’s sleep duration, suggesting that parental HL may be an appropriate target for interventions aiming to lengthen children’s sleeping time.

## 1. Introduction

Short sleep duration has been linked to a wide array of poor mental and physical health outcomes [1]. In children, sleep has a significant influence on developmental outcomes and is commonly overlooked as an ecological influence on their development [2]. Previous studies suggested that the shortening of nocturnal sleep is associated with cardiac autonomic hypofunction and low systolic blood pressure in preschool children [3,4]. In addition, the poor sleep schedules of preschool-aged children were found to predict behavioral problems during the primary school years [5]. Sleep problems of 5–6-year-olds have been associated with poor behavior, obesity and negative secondary effects on maternal and family well-being [6]. Collectively, sleep in preschool children is an important factor for their health and active lives.

Adequate sleeping time for children has not been fully elucidated but the National Sleep Foundation (NSF) in the US proposed sleep duration recommendations for preschool-aged children (3–5 years) of 10–13 h [7]. In contrast, the lack of sleep in preschool children is a serious public health problem in Japan. A previous study in Japan showed that the percentage of children aged 3.5 years old who slept for less than 10 h was 39.5% [8]. Another study in Japan suggested that the percentage of preschool children who went to sleep before 9 p.m. was only around 15% (3 years old: 14.6%, 4 years old: 16.9%, 5–6 years old: 12.9%) [9], suggesting insufficient sleep duration in the majority of Japanese preschoolers. It is interesting to note that children’s bedtime correlated with their parent’s bedtime in Japan [10]. This study suggested that parents’ inadequate sleeping and life habits were associated with their children’s insufficient sleep duration. Thus, parents can have a great influence on their children’s sleep duration.

The relation between health literacy (HL) and health status is now well recognized and better understood [11]. HL is defined as a person’s ability to access, understand, evaluate, communicate and use health information to make decisions for one’s health [12]. According to previous studies, an adult’s low HL has been associated with their own insufficient health status and insufficient health outcomes [13,14] and parents’ low HL has also been associated with insufficient health outcomes for their children [15,16,17,18,19]. Previous studies in Japan suggested that the people with higher HL were positively associated with adult healthy lifestyle characteristics [20] and that people with insomnia were less likely to seek help for their problem [21].

Improvement of parents’ HL encourages improvement of their own lives and sleep habits and it may help to improve the sleep duration of their children. Thus, it is important to investigate the association between parents’ HL and their children’s sleep duration in Japan. Although one study in the US investigated the association between parents’ HL and children’s sleep issues [22], due to the lack of knowledge on this subject in Japan, no consensus has been reached on parents’ HL and their children’s sleep issues. Therefore, the hypothesis of the present study was that the low HL of parents would be associated with the insufficient sleep duration of their children. The purpose of this study was to investigate the association between the sleep duration of preschool children and the HL of their parents in Japan.

## 2. Methods

### 2.1. Participants

This was a multicenter cross-sectional cohort study using a convenience sample. The participants included preschool children (3–6 years old) and their parents who lived in Chitose city, Hokkaido, Japan. They attended kindergarten, nursery school, or a center for early childhood education and care: two kindergartens, one nursery school and one center for early childhood education and care. To invite them to participate, we asked the school staff to distribute materials that included the intention of the study, the consent for participation in the study, the withdrawal of consent to participate and the questionnaire to parents in September 2016. If the parents had more than one child in the same school, we asked them to answer the questionnaire for their oldest child. We excluded parents for whom a questionnaire had missing values. Participants voluntarily provided written informed consent. This study received prior approval from the Research Ethics Committee of Kobe University (approval number 498).

### 2.2. Demographic and Socioeconomic Characteristics

The following demographic and socioeconomic characteristics were investigated: parent sex; parent age; parent height and weight; parent alcohol and smoking behavior; marital status, married or not; education level (up to high school, 2-year college or vocational college, college graduate or above); household income level (<5 million yen, ≥5 million yen); child age; child birthweight; and number of siblings.

### 2.3. Health Literacy of Parents

We assessed the HL of parents with the 14-item Health Literacy Scale (HLS-14) questionnaire, which was created in Japan (Table 1) [23]. A previous study on the HLS-14 indicated that it is an adequate questionnaire with which to evaluate comprehensive HL [24]. Every question is estimated at 5 stages. The total score is calculated by totaling the scores of all answers. The scores range from 14 to 70 and were analyzed as continuous variables. The higher the score, the better the comprehensive HL is. The validity and reliability of this method was already shown [23]. We divided the participants into two groups according to an HLS-14 score >50 (high HL group) and an HLS-14 score <50 (low HL group) [23,25].

### 2.4. Children’s Sleeping Duration

Although sleep duration is a continuous variable, it can be difficult for participants to provide the time in minutes and seconds. Therefore, we asked the parents to provide their average children’s sleeping hours and minutes per day during the previous one month.

### 2.5. Statistical Analysis

The differences in the clinical characteristics between the low and the high HL groups were determined by the Student *t*-test or Mann-Whitney U test. Pearson correlation analysis was performed to compare the parents’ HL and the children’s sleeping times. The median HL score of the HLS-14 in Japanese adults was 50 points [23]. Furthermore, the trend of the children’s sleeping time was greatly different at 50 points or less. As such, to investigate the parent’s HL and their child’s sleeping time in detail, we divided the parents’ HL into two groups according to a score above or below 50 points on the HLS-14 and analyzed children’s sleeping time separately. All statistical analyses were performed using EZR (Saitama Medical Center, Jichi Medical University, Saitama, Japan), which is a graphical user interface for R (The R Foundation for Statistical Computing, Vienna, Austria). Differences and correlations were considered significant when *p* < 0.05.

## 3. Results

**Figure 1:**
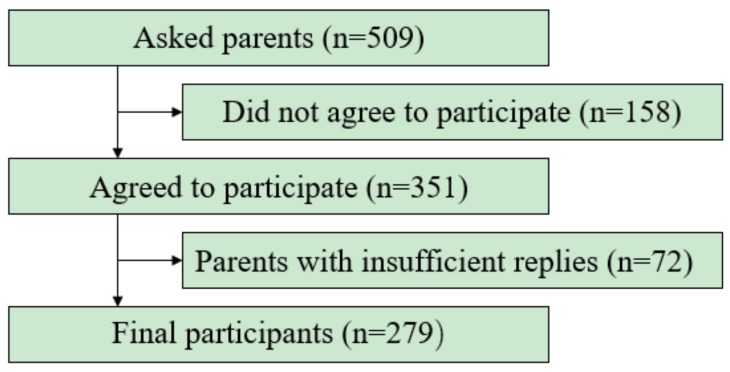
Chart of participant flow during the study.

Figure 1 shows participant flow during this study. The original response rate of the target population was 68.9% (351/509) but after the 72 parents were excluded, the final response rate was 54.8% of the target population).

Table 2 shows the demographic differences between the low HL group and high HL group. The number of participants in the high HL group was 210 (75.3%) and that in the low HL group was 69 (24.7%). The average sleep duration of the children was 9.4 ± 0.9 h and it was significantly longer in the high HL group (9.5 ± 0.9 h) than in the low HL group (9.1 ± 1.1 h) (*p* = 0.01). In addition, the number of siblings (*p* = 0.03), parent’s age (*p* = 0.05) and the percentage of females (*p* = 0.01) were greater in the high HL group than in the low HL group.

**Figure 2:**
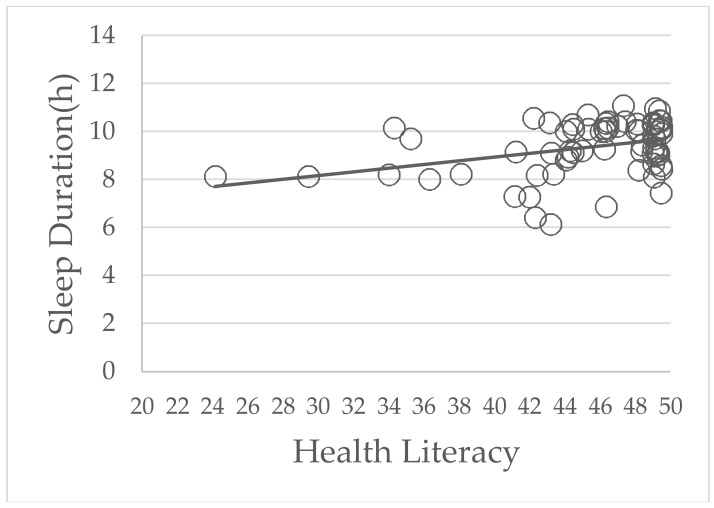
The correlation between parents’ health literacy and their children’s sleep duration in the low health literacy group. The *p* value was 0.01 and the correlation coefficient (r) was 0.32.

**Figure 3:**
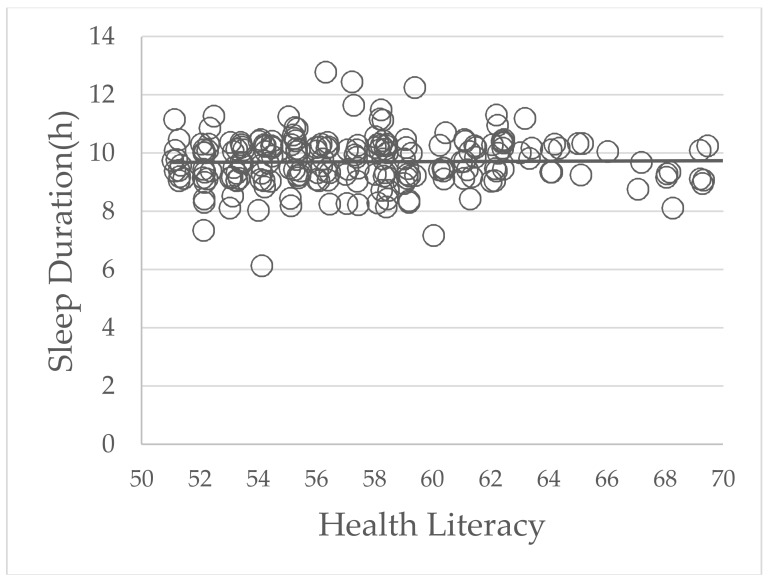
The correlation between parents’ health literacy and their children’s sleep duration in the high health literacy group. The *p* value was 0.98 and the correlation coefficient (r) was 0.0009.

The correlation between parents’ HL and children’s sleeping times is shown in Figure 2 and Figure 3. In the low HL group, a positive correlation was shown between the parents’ HL and children’s sleeping times (*p* = 0.01, r = 0.32). However, in high HL group, there were no statistically significant differences between parents’ HL and children’s sleeping times (*p* = 0.98, r = −0.0009).

## 4. Discussion

This is the first study, to our knowledge, to show that parents’ comprehensive HL correlated positively with the sleep duration of their children in Japan. Moreover, this correlation was observed only in the low HL group. A similar study in the US suggested that if the parents’ functional HL was low, the sleep duration of their children was likely to be short [22]. In the Japanese population, even though the level of functional HL is high [24], comprehensive HL is relatively lower than that of other populations [26]. In the present study, comprehensive HL was assessed with the HLS-14, so this study should have great significance in Japan.

There are three possible explanations for the observed associations between parents’ HL and their child’s sleep duration in this study. First, Japanese parents with low HL may not be able to access health and medical professionals to obtain the appropriate health information of their children. One study suggested that those with low (vs. adequate) HL were significantly less likely to access health information about their children [27]. Second, Japanese parents with low HL may not be able to understand the importance of sleep for their children. Parents, particularly pregnant and new mothers, rely on obstetric and pediatric providers, along with parenting books and online sources, for information on common parenting questions, especially with respect to their children’s sleep [28]. Another study suggested that parents with low HL also have greater difficulty understanding and acting on health recommendations [14,18]. Thus, if the parents’ HL is low in Japan, they may less likely to understand the health information pertaining to their children. Third, parenting behavior may be associated with the sleep duration of children in Japan. It was already proven that parental intervention positively influenced developmentally appropriate bedtime routines, sleep-related behaviors and sleep duration of infants [29]. Another study indicated that low parent HL has been linked to poor parenting practices and to inadequate parenting behavior such as putting a TV in the room where their child sleeps [22]. Lo suggested that it was easy for preschool-aged children without a bedtime TV-viewing habit to have an adequate quantity of sleep [30]. Thus, we believe that low HL in some Japanese parents may result in poor parenting, which may then be associated with the sleep duration of their preschool children. However, we did not investigate variables relevant to childhood development, sleep quality and individual family patterns of sleep behaviors, which are significant factors for sleep in children and the issue of poor parenting. Therefore, additional study is needed to investigate this association in the future.

The results of the present study are not consistent with other previous studies that showed significant relationships between the low HL of parents and their own insufficient sleep duration [21], high body mass index (BMI) and drinking and smoking behaviors [31,32]. A possible explanation is that most (92.4%) of the participating parents in the present study were mothers. An earlier study suggested that the HL of Japanese women was not associated with their healthy lifestyle characteristics, such as alcohol consumption, smoking behavior, exercise frequency, obesity (BMI), sleep duration, breakfast and snacking between meals [20]. Also, the results of the present study are not consistent with other previous studies that showed significant relationships between the low HL of parents and their own low educational level. A possible explanation for this is that the proportion of parents whose education was at high school or 2-year college level was extremely high (81.7%). Moreover, we regarded education level as a continuous variable in our analysis. Consequently, we found no significant relationships between the low HL of the parents and their own health behaviors such as those listed above and their own low educational level.

However, parents’ sex and age and the number of siblings were associated with the parents’ HL. There are two possible explanations for these observed associations with parents’ sex. First, we think the difference is related to the material used by men or women to obtain health information. A previous study suggested that more women get nutritional information from books/magazines or from health professionals, whereas more men get this information from their friends [33]. Second, it is easier for mothers than fathers to have good relations with their neighbors, with whom they can talk about health. In Japan, almost all domestic work and child care is performed by mothers and there are almost no cases of a father doing such tasks. Therefore, we think that it is easier for a mother than a father to form relationships in which information on the health of their children can be shared with their friends or neighbors. The reason that the HL of young parents was significantly higher than that of older parents relates to the greater percentage of men in the low HL group than in the high HL group. In 2010, the average age of first marriage was 30.5 years in Japanese men and 28.8 years in Japanese women. So, the age of the men in the low HL group, which had a proportionately greater number of men, was high. Finally, we consider the reason for the association with the number of siblings. In Chitose, every child undergoes five yearly checkups, from years 1 to 5, so parents with more than one child have more chances to obtain information about their children’s health. As well, when they raise more than one child, they can put the knowledge and methods learned to resolve various health problems.

According to Figure 2 and Figure 3, parents’ higher HL scores were associated with longer sleep duration of their children only in the low HL group. A previous study showed that Japanese adults with lower HLS-14 scores were significantly less likely to recognize problems such as the risk of illness, the need for preventive action and the reluctance to take preventive action in compliance with their doctor’s advice [25]. In other words, parents with low HL were less likely to understand and use health information to make decisions for their own or their children’s health. In contrast, parents with high HL were more likely to have these abilities. Consequently, parents’ HL correlated positively with children’s sleep duration only in the low HL group. This result suggested that to improve the sleep duration of preschool children, parents with low HL need to be open to all interventions. However, the positive correlation between parents’ low HL and the reduced sleep duration in their children was weak (r = 0.32; r < 0.4, weak; r ≥ 0.4 but r < 0.6, moderate; r ≥ 0.6 but r < 0.8, strong [34]). Therefore, there is a need for further studies to confirm our findings in a larger population and to determine a causal relationship between parents’ HL and their children’s sleep duration.

There are some limitations in this study. First, this is a cross-sectional study and thus, we cannot determine the causality of various factors. Second, we collected data in only one small area of Japan, so the results obtained may not apply to other parts of Japan or the world. Third, the sample size was small, so we did not perform multivariate analysis to adjust for potential confounding factors. Moreover, we did not detect cut off points of HL. Fourth, The HLS-14 has been used for an adult population in previous studies, not for an adult parent population. Fifth, it could be possible that the parents who did not agree to participate had relatively had low HL compared with those who did participate. Sixth, previous studies suggested that long naps affect nighttime sleep patterns [35,36] and the quality of sleep [37]. Not only the total sleeping time but also the quality of the sleep is important [38,39,40,41,42]. However, we did not collect information relevant to childhood development, sleep quality and individual family patterns of sleep behavior, which are significant factors for the sleep of children and the issue of poor parenting. Also, we did not investigate factors that are important variables relating to childhood development, sleep quality, sleep behaviors, sleep duration such as parents’ working patterns, dwelling and residential conditions, location of sleep of the children, parent and child health, physical activity and screen time. Seventh, one study suggested that parents tended to overestimate the sleep duration of their children [43] and this study used a parent-reported questionnaire. Eighth, an important variable that relates to sleep duration would also include sleep location of the child but we did not specifically investigate this.

Finally, it is important to go to sleep at a regular time [10,44,45,46] but we also did not collect this information. Therefore, even though the duration of sleep may be long, the quality of the sleep is unknown.

## 5. Conclusions

There was a statistically significant difference between parents’ HL and the sleep duration of their preschool children. A weak positive correlation between parents’ HL and their children’s sleep duration was observed only in the low HL group. These results suggested that parental HL may be an appropriate target for interventions aiming to lengthen children’s sleeping time. In the future, prospective and longitudinal studies to investigate the clinical effects of parents’ HL will be warranted.

## Figures and Tables

**Table 1:** The 14-item Health Literacy Scale.

When you read instructions or leaflets from hospitals or pharmacies, how do you agree or disagree about the following?						
		Strongly Disagree	Disagree	Not Sure	Agree	Strongly Agree
1	I find characters that I cannot read	5	4	3	2	1
2	The print is too small for me (even though I wear glasses)	5	4	3	2	1
3	The content is too difficult for me	5	4	3	2	1
4	It takes a long time to read them	5	4	3	2	1
5	I need someone to help me read them	5	4	3	2	1
If you are diagnosed as having a disease and you have little information about the disease and its treatment, how to you agree or disagree about the following?						
		Strongly Disagree	Disagree	Not sure	Agree	Strongly Agree
6	I collect information from various sources	1	2	3	4	5
7	I extract the information I want	1	2	3	4	5
8	I understand the obtained information	1	2	3	4	5
9	I tell my opinion about my illness to my doctor, family or friends	1	2	3	4	5
10	I apply the obtained information to my daily life	1	2	3	4	5
If you are diagnosed as having a disease and you can obtain information about the disease and its treatment how do you agree or disagree about the following?						
		Strongly Disagree	Disagree	Not sure	Agree	Strongly Agree
11	I consider whether the information if applicable to me	1	2	3	4	5
12	I consider whether the information is credible	1	2	3	4	5
13	I check whether the information is valid and reliable	1	2	3	4	5
14	I collect information to make my healthcare decisions	1	2	3	4	5

Table taken from Ref. [23].

**Table 2:** Characteristics Differences between the High Health Literacy and Low Health Literacy Groups.

Characteristic	Total (*n* = 279)	High Health Literacy Group (*n* = 210)	Low Health Literacy Group (*n* = 69)	*t*-Value or χ^2^ Value *	*p* Value
Child					
Age (months)	56.4 ± 10.0	56.3 ± 9.9	56.6 ± 10.6	−0.15	0.88
Sex (girls)	119 (42.7%)	89 (42.3%)	30 (43.4%)	0.15 *	0.87
BMI (kg/m^2^)	15.6 ± 1.3	15.5 ± 1.2	15.7 ± 1.6	−0.65	0.52
Birth weight (g)	3012.0 ± 451.1	2995.3 ± 431.8	3063.7 ± 506.2	−1.09	0.28
Number of siblings (n)	1.2 ± 0.8	1.3 ± 0.8	1.1 ± 0.8	2.20 *	0.03
Sleep duration (Continuous h)	9.4 ± 0.9	9.5 ± 0.9	9.1 ± 1.1	2.50	0.01
Breakfast (everyday eater)	261 (93.6%)	199 (94.8%)	62 (89.6%)	1.47 *	0.14
Parent					
Age (years)	36.2 ± 5.1	35.9 ± 5.2	37.2 ± 4.9	−2.02	0.05
Sex (female)	258 (92.4%)	199 (94.7%)	59 (85.5%)	2.58 *	0.01
BMI (kg/m^2^)	21.1 ± 2.9	21.1 ± 2.8	21.1 ± 3.1	−0.19	0.85
Marital status (married)	258 (92.4%)	195 (92.8%)	63 (91.3%)	0.70 *	0.50
Education (years)	13.3 ± 1.6	13.3 ± 1.6	13.2 ± 1.7	−0.59	0.56
Household income <5 million Yen (n)	143 (51.3%)	111 (52.9%)	32 (46.4%)	0.93 *	0.35
Alcohol consumption (non-everyday drinker)	255 (91.4%)	193 (91.9%)	62 (89.9%)	1.02 *	0.31
Smoking behavior (non-smoking)	225 (80.6%)	170 (81.0%)	55 (79.7%)	0.22 *	0.82
Sleep duration (h)	6.4 ± 1.1	6.4 ± 1.2	6.4 ± 1.1	−0.53	0.60

The study subjects were divided into two groups according to a total health literacy score of above or below the cutoff value of 50. Data are expressed as mean ± standard deviation or number (percentage). BMI, body mass index.

## Abbreviations

CI	confidence interval
HL	health literacy
HLS-14	14-item Health Literacy Scale
NSF	National Sleep Foundation
